# Influence of Nonlinear Effects Induced by Mode Coupling on Vibration Trajectories of MEMS Micromirrors

**DOI:** 10.3390/mi16060723

**Published:** 2025-06-19

**Authors:** Zhen Chen, Dayong Qiao, Anjie Peng

**Affiliations:** Ministry of Education Key Laboratory of Micro/Nano Systems for Aerospace, Key Laboratory of Micro- and Nano-Electro-Mechanical Systems of Shaanxi Province, School of Mechanical Engineering, Northwestern Polytechnical University, 127 Youyi West Road, Xi’an 710072, China; chenzhen407@mail.nwpu.edu.cn (Z.C.); penganjie@mail.nwpu.edu.cn (A.P.)

**Keywords:** micromirror, mode coupling, vibration trajectory

## Abstract

Detection of the vibration trajectories of MEMS micromirrors is crucial for ensuring their application performance. This study investigates key factors influencing micromirror vibration trajectories. When actuated by a square-wave signal containing high-frequency components, micromirrors exhibit mode coupling vibrations. By incorporating a mode coupling mechanism, this paper establishes a comprehensive vibration trajectory model for micromirrors. Numerical simulations were performed to obtain trajectory solutions. Both the experimental and simulation results demonstrate that the mode coupling leads to deviations between the actual trajectory and the expected sinusoidal pattern. These deviations compromise the accuracy of trajectory prediction systems, which typically assume that the trajectory follows a sinusoidal pattern. To mitigate the deviations caused by mode coupling, this study proposes structural parameter optimization during the micromirror design process.

## 1. Introduction

MEMS micromirrors manipulate the direction of reflected light beams through rotational vibration of the mirror plate. MEMS micromirrors offer significant advantages, such as a small size, light weight, low cost, and low power consumption. They are widely used in various fields, including display projection [1], LiDAR [2,3], and 3D cameras [4].

In micromirror applications, vibration trajectories are critical operational parameters that determine the accuracy of reflected light beam adjustment [5,6]. Consequently, detection systems are essential for monitoring trajectories. Such detection systems integrate high-speed photo-diodes along the reflected light beam path. Since micromirror vibration trajectories are conventionally assumed to follow a sinusoidal pattern, the detection systems record the time intervals of the reflected light beam reaching the photo-diodes to reconstruct sinusoidal trajectories as real-time vibration trajectories [7]. However, experimental observations reveal deviations between the actual vibration trajectories and the expected sinusoidal patterns. The deviations arise from higher harmonics in the trajectories, and these higher harmonics originate from mode coupling within vibration systems like micromirrors [8].

Mode coupling in vibration systems has attracted widespread attention due to the complex nonlinear behavior inherent in dynamic models [9,10,11]. Mode coupling contributing factors include structural parameters [12,13], material properties [14], environmental conditions [15,16,17], and driving signals [18,19]. For micromirrors actuated by multi-frequency drive signals, the signals usually serve as the primary contributor of the mode coupling [20,21,22,23]. When the natural frequencies of higher-order modes exceed the frequency ranges of the driving signals, structural geometric nonlinearity becomes the dominant mode coupling factor [24,25,26,27,28,29]. Most existing studies have focused on establishing mode coupling dynamic models to analyze the impacts on the frequency domain [30,31]. Although some researchers have derived time-domain vibration trajectories through torsional-mode dynamic models, these studies neglect the mode coupling influences [32,33]. Collectively, current research fails to adequately explain deviations between observed vibration trajectories and expected sinusoidal patterns.

This study investigates the impact of mode coupling on torsional-mode vibration trajectories. The paper is organized as follows: In Section 2, the modes of the MEMS micromirror are analyzed, and the coupled modes during the vibration process are identified. In Section 3, a torsional-mode dynamic model incorporating the mode coupling nonlinearity is developed using continuum mechanics principles. In Section 4, the simulated vibration trajectories are compared with the experimental results for validating the model. Finally, based on these findings, a method to reduce vibration trajectory deviations is proposed.

## 2. Micromirror Mode Analysis

**Figure 1:**
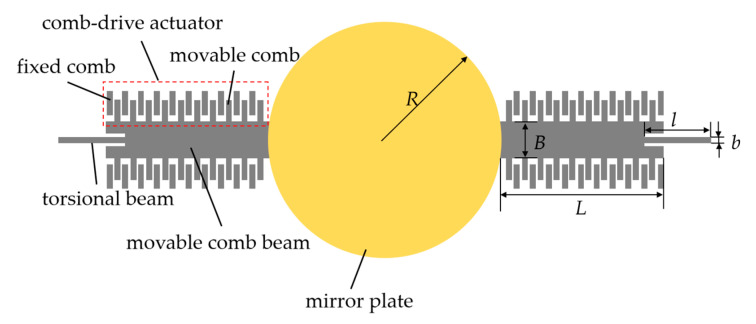
Schematic diagram of the main structures of a micromirror.

The micromirror analyzed in this study is fabricated from a silicon-on-insulator (SOI) wafer. As shown in Figure 1, the primary structures of a micromirror comprise a mirror plate, four symmetrically arranged comb-drive actuator pairs, and a set of torsional beams. Two movable comb beams of the comb-drive actuators are connected to the mirror plate as a rigid body. The rigid body is anchored via two torsional beams. Electrically, the movable combs interface with drive electrodes, while the fixed combs are grounded. The key structural parameters of the micromirror and the corresponding values are listed in Table 1. The micromirror is micromachined from silicon with material properties defined as follows: Young’s modulus E = 128 GPa, Poisson’s ratio ν = 0.22, and density ρ = 2230 kg/m^3^.

**Figure 2:**
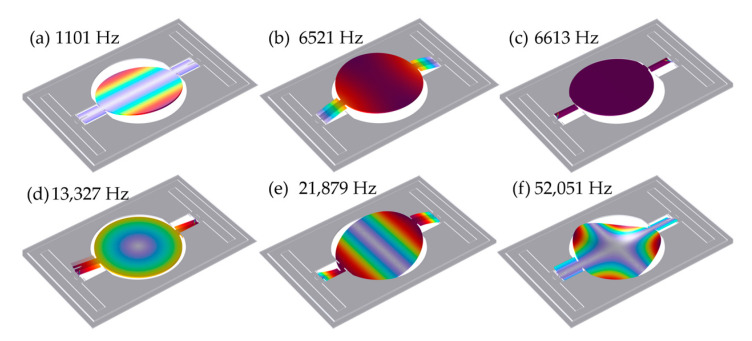
The first six modes of MEMS micromirrors.

To achieve higher vibration amplitudes, the micromirror is actuated into resonance. The square-wave signal is chosen to drive the micromirror due to its high power density capability and simple drive circuitry [34]. The square-wave signal is applied to the drive electrode to induce alternating electrostatic forces. The resulting electrostatic forces drive the mirror plate into torsional resonance about the torsional beams. The micromirror exhibits higher-order modes in addition to the primary torsional mode; the first six modes are shown in Figure 2.

COMSOL Multiphysics ver.6.2 simulation software is used to obtain the natural frequencies of the vibration modes. The natural frequency for each mode as shown in Figure 2 is found using eigenfrequency analysis. As quantified in Figure 2a, the natural frequency of the torsional mode for the micromirror is 1101 Hz. To drive torsional resonance, the square-wave signal is set to twice the torsional-mode natural frequency. The Fourier series expansion of the square-wave signal reveals a fundamental frequency component at 2200 Hz, along with high-frequency components at odd integer n multiples (n = 3, 5, 7,…). The natural frequencies of the out-of-plane bending mode in Figure 2b and the in-plane bending mode in Figure 2c are 6521 Hz and 6613 Hz, respectively. The natural frequencies of the higher-order bending modes align with the high-frequency component (n = 3) of the square-wave signal and are therefore excited. The natural frequencies of the remaining higher-order modes in Figure 2d–f fall outside the spectral range of the driving signal and remain unexcited.

In summary, the square wave excites mode coupling among the first three vibration modes of the micromirror. Given the presence of mode coupling, a torsional-mode dynamic model incorporating the coupling effect must be developed. This model enables simulation of a more precise vibration trajectory and explains the deviation between the observed trajectory and the ideal sinusoidal pattern.

## 3. Vibration Trajectory Model Construction

When coupled-mode vibration occurs in the micromirror, the torsional beam deformation involves coupled in-plane bending, out-of-plane bending, and torsional displacement, resulting in nonlinear geometric deformation. Due to the mode coupling effects, the stress–strain relationship of the torsional beam does not satisfy the linear elastic assumption. Therefore, mode coupling introduces nonlinear terms into the vibration equations for in-plane bending, out-of-plane bending, and torsional modes [35]. The vibration equations for each mode of the micromirror are established using Hamilton’s principle in continuum mechanics. The primary objective of this paper is to analyze the mode coupling term in the vibration equations, and develop an expression for the nonlinear term based on the eigenfunctions of vibration equations. The working mode is the torsional mode. Consequently, the nonlinear term induced by the mode coupling effect is incorporated into the existing torsional-mode vibration equation to develop a trajectory model, enabling an accurate numerical solution of the vibration trajectory.

**Figure 3:**
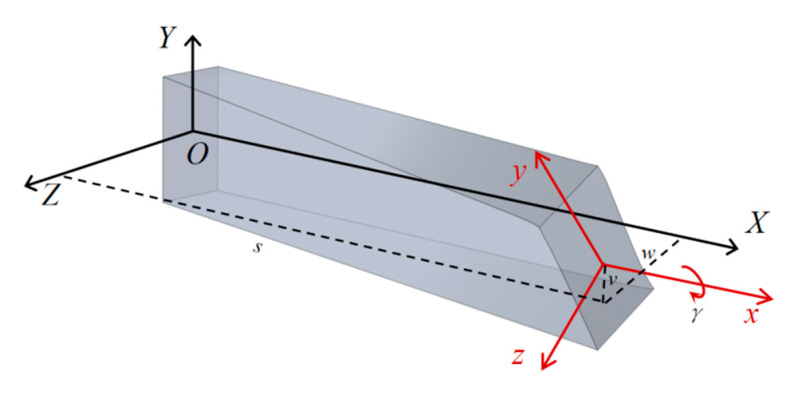
Coordinate systems used in the development of motion equations.

The nonlinear term in the torsional-mode vibration equation is inherently related to the eigenfunctions of both in-plane and out-of-plane bending vibration equations. The analysis begins with deriving the bending-mode vibration equations for the integrated rigid body. As shown in Figure 3, the in-plane and out-of-plane displacements of the torsional beam cross-section are defined as u(s, t) and v(s, t), respectively, where s denotes the length coordinate from the anchor point. At s = L, u and v correspond to the displacements of the integrated rigid body.

The Lagrangian function of the micromirror is formulated by incorporating the kinetic energy, strain energy, and external electrostatic energy [31]. Using the Lagrangian function, the Euler–Lagrange equations can be developed. Applying Hamilton’s principle, the bending-mode vibration equations are derived through variational calculus on the Euler–Lagrange equations. Based on the Galerkin method, by solving the linearized undamped bending-mode vibration equations, the linear eigenfunctions F_v_, F_w_ and eigenfrequencies ω_v_, ω_w_ are obtained. The eigenfunctions F_v_ and F_w_ represent the bending mode shapes of the micromirror, corresponding to the displacement distribution of the micromirror when vibrating at the eigenfrequencies ω_v_ and ω_w_. The expressions for F_v_ and F_w_ are as follows:

(1) $$F_{i}=\cosh\left(r_{i1}s\right)-\cosh\left(r_{i2}s\right)-K_{i}\left[\sinh\left(r_{i1}s\right)-\frac{r_{i1}}{r_{i1}}\sinh\left(r_{i2}s\right)\right],\left(i=v,w\right)$$

In order to solve the bending-mode eigenfunctions, the eigenvalues r_k1_ and r_k2_ are required first. r_k1_ and r_k2_ in Equation (1) are obtained by solving the following characteristic equation:

(2) $$r_{i1}^{4}+r_{i2}^{4}+2r_{i1}^{2}r_{i2}^{2}\cosh\left(r_{i1}\right)\cos\left(r_{i2}\right)+r_{i1}r_{i2}\left(r_{i2}^{2}-r_{i1}^{2}\right)\sinh\left(r_{i1}\right)\sin\left(r_{i2}\right)=0$$

In the vibration analysis of the micromirror torsional mode, the boundary conditions at the fixed end require that the functions and their derivatives be zero, while the boundary conditions at the other end require that the higher-order derivatives of the eigenfunctions be zero. By imposing the boundary condition $F_{v,w}^{\prime \prime \prime }=0$ on Equation (1), the variables r_i1_ and r_i2_ (i = v, w) are

(3) $$r_{v1}=\sqrt{-\frac{J_{w}D_{v}\omega_{v}^{2}}{2D_{w}}+\sqrt{{\left(\frac{J_{w}D_{v}\omega_{v}^{2}}{2D_{w}}\right)}^{2}+\frac{D_{v}\omega_{v}^{2}}{D_{w}}}}$$

(4) $$r_{v2}=\sqrt{\frac{J_{w}D_{v}\omega_{v}^{2}}{2D_{w}}+\sqrt{{\left(\frac{J_{w}D_{v}\omega_{v}^{2}}{2D_{w}}\right)}^{2}+\frac{D_{v}\omega_{v}^{2}}{D_{w}}}}$$

(5) $$r_{w1}=\sqrt{-\frac{J_{v}\omega_{w}^{2}}{2}+\sqrt{{\left(\frac{J_{v}\omega_{w}^{2}}{2}\right)}^{2}+\omega_{w}^{2}}}$$

(6) $$r_{w1}=\sqrt{\frac{J_{v}\omega_{w}^{2}}{2}+\sqrt{{\left(\frac{J_{v}\omega_{w}^{2}}{2}\right)}^{2}+\omega_{w}^{2}}}$$

Here, J_j_ (j = v, w) denotes the distributed mass moments of inertia of the rectangular section, and D_v_ and D_w_ are the principal flexural stiffnesses of the torsional beam. The introduction of the coefficient K ensures that these boundary conditions are satisfied, thereby accurately describing the vibration modes under different boundary conditions. And the formulas for K_v_ and K_w_ are, respectively,

(7) $$K_{i}=\frac{r_{i1}^{2}\cosh r_{i1}+r_{i2}^{2}\cosh r_{i2}}{r_{i1}^{2}\sinh r_{i1}+r_{i2}^{2}\sinh r_{i2}},\left(i=v,w\right)$$

The nonlinear torsional-mode dynamic influencing the vibration trajectory originates from the geometric nonlinearity induced by mode coupling effects. In the torsional-mode vibration equation, the bending-mode eigenfunctions are projected onto the torsional mode via integration, introducing a quadratic nonlinear term into the vibration equation [31,35]. The coefficient of the quadratic nonlinear term is derived as

(8) $$k_{1}=\frac{D_{w}-D_{v}}{D_{w}}\int_{0}^{L}\left(\sqrt{2}\sin(\frac{\pi }{2}s)F_{v}^{\prime \prime }F_{w}^{\prime \prime }\right)ds$$

Substituting the quadratic nonlinear term, the vibration trajectory with torsional angle γ in the torsional-mode vibration equation, in which the mode coupling effect is considered, can be obtained as

(9) $$J\ddot{\gamma }+c\dot{\gamma }+k\gamma +k_{1}\gamma^{2}=-{U_{m}}^{2}b_{0}b_{1}\gamma e^{-b_{1}\gamma^{2}}$$

Here, J is the inertia around the torsional axis, c denotes the damping coefficient, k denotes the torsional stiffness coefficient, and k_1_ denotes the nonlinear coefficient induced by the modal coupling. The inertia J, torsional stiffness k, and damping coefficient c can be calculated by simulation software. For the electrostatic torque on the right side of Equation (9), it is necessary to calculate the relationship curve between the capacitance and the torsional angle γ using finite element simulation software. Next, the relationship curve is differentiated with respect to the twist angle γ. The differentiated result is combined with the drive signal voltage to obtain the coefficients b_0_ and b_1_ for the electrostatic torque expression [36]. Therefore, b_0_ and b_1_ are the fitting parameters for capacitance and the torsional angle γ function. U_m_ denotes the voltage amplitude of the square-wave signal with a duty cycle of 50%, which is inputted to the comb-drive actuators. By simulation modeling, the numerical solution of the vibration trajectory can be obtained. The key parameters of the torsional-mode vibration equation studied in this paper are shown in Table 2 below.

## 4. Simulation and Experiment Results

### 4.1. Simulation

To validate the torsional-mode vibration equation, a numerical simulation was performed to compute vibration trajectory numerical results for direct comparison with the experimental results. A SIMULINK-based torsional-mode dynamic model was developed to output the vibration trajectory of the micromirror.

The model employed a 120 V square wave at a 50% duty cycle. The signal frequency was adjusted to modulate the trajectory amplitude between 6° and 16°. This approach enables analysis of mode-coupling-induced nonlinear effects across varying amplitudes. The simulation results are compared with the experimental results, as detailed in the following subsection.

### 4.2. Experiment

**Figure 4:**
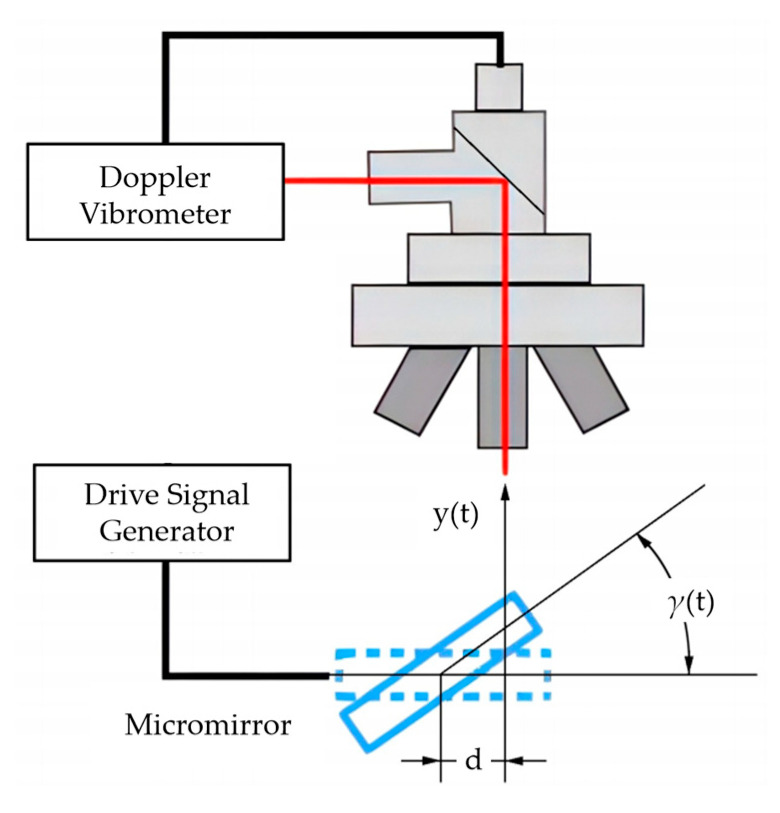
Experimental setup for measuring vibration trajectory.

The experimental setup is illustrated in Figure 4. The key experimental instruments were a Laser Doppler Vibrometer (LDV) and a driving signal generator.

The LDV projected a micron-scale laser spot onto the mirror plate at a distance d from the torsional beam center to measure the out-of-plane displacement y(t). The vibration trajectory data was reconstructed by processing the collected discrete displacement data y(t), which was transformed via trigonometric transformation. The torsional angle γ(t) of the vibration trajectory was defined by the displacement y(t) using the following equation:

(10) $$\gamma \left(t\right)=\arctan\left(\frac{y\left(t\right)}{d}\right)$$

The distance d = 130 μm was selected to balance two critical requirements: maximizing measurement sensitivity and preventing signal distortion. A smaller d enhances the signal-to-noise ratio of the torsional angle measurement due to Equation (10). Simultaneously, this specific distance ensures the out-of-plane displacement remains well within the linear operating range of the LDV across all tested vibration amplitudes, effectively preventing signal distortion at maximum deflection. This setting achieves an optimal compromise between measurement precision and measurement range.

**Figure 5:**
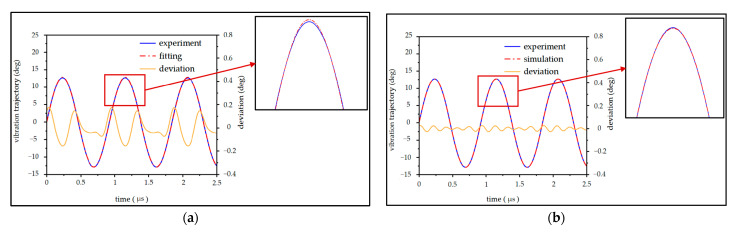
Comparison of the vibration trajectory experimental result with (**a**) the ideal sinusoidal fitting curve and (**b**) the numerical result of the simulation.

Consistent with the simulation configuration, the driving signal was a 120 V square wave with a 50% duty cycle. The actual vibration trajectory obtained via the experimental setup described above is compared with an ideal sinusoidal fit pattern in Figure 5a. In Figure 5b, the simulated vibration trajectory is further compared with the actual experimental result.

**Figure 6:**
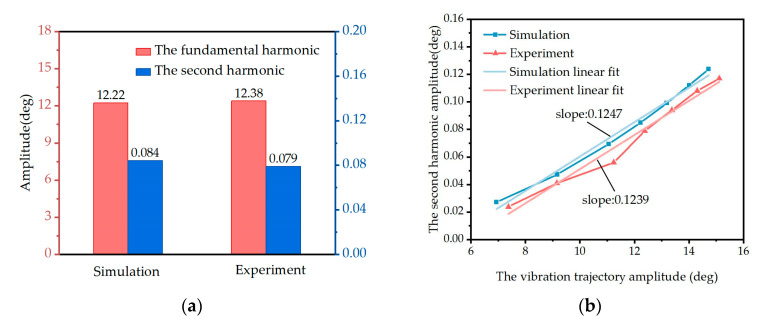
(**a**) The amplitude of the fundamental harmonic and second harmonic of the simulation and experiment results. (**b**) The linear amplitude relationship between the vibration trajectory and the second harmonic.

Compared with the sinusoidal pattern, the simulation model’s numerical solution shows closer agreement with the actual vibration trajectory. In Figure 6a, frequency-domain analysis of both the experimental and simulation results reveals that the observed deviations originate from the presence of a second harmonic component. The second harmonic component stems from the quadratic nonlinear term in the torsional-mode vibration equation. The frequency-domain analysis demonstrates good consistency between the experimental and simulation results.

Through adjustment of the driving signal frequency, the vibration trajectory amplitude was controlled within the range of 6° to 16°. As shown in Figure 6b, within the above amplitude range, the second harmonic component amplitude exhibits a proportional relationship with the vibration trajectory amplitude, confirming the amplitude-dependent enhancement of nonlinear effects induced by mode coupling. The concordance between the simulated and experimental trends validates the inclusion of mode coupling nonlinearity in the torsional-mode vibration equations.

## 5. Mode Coupling Nonlinearity Effect Mitigation via Structural Parameter Optimization

Due to the identified mode coupling, a quadratic nonlinear term k_1_ is introduced into the torsional-mode vibration equation. And the quadratic nonlinear term k_1_ is the primary source of the second harmonic distortion observed in the vibration trajectory. Analysis of the quadratic nonlinear coefficient in Section 3 has revealed an inherent correlation between the coefficient magnitude and the geometric parameters of the micromirror. For the micromirror in this study, the torsional beam length and the rectangular section aspect ratio were selected for the correlation analysis.

**Figure 7:**
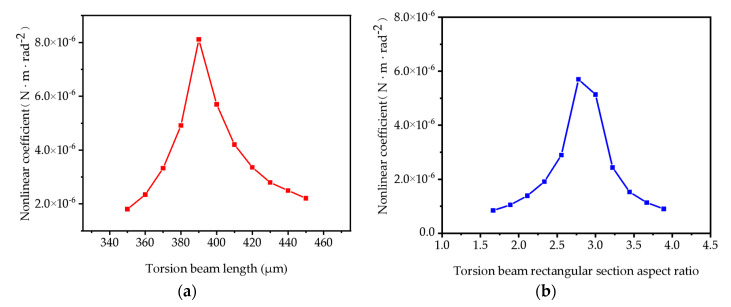
(**a**) Relationship between torsion beam length and nonlinear coefficient magnitude; (**b**) relationship between torsion beam rectangular section aspect ratio and nonlinear coefficient magnitude.

From Figure 7, it can be seen that there are extreme value points in the functional of the quadratic nonlinear coefficient with respect to the geometric parameters. Figure 7a plots k_1_ against the length of the torsion beams. The curve reveals a distinct maximum value near l = 390 μm. Figure 7b plots k_1_ against the aspect ratio b/h of the torsion beam rectangular section. This curve also exhibits a clear maximum point near b/h = 2.7. A smaller k_1_ directly reduces the amplitude of the second harmonic component in the vibration trajectory, thereby minimizing deviations from the ideal sinusoidal pattern.

The dependence of k_1_ on geometric parameters provides a powerful design approach to mitigating mode coupling and its nonlinear effects on the vibration trajectory. The physical mechanism enabling this minimization is the shifting of all the modes’ natural frequencies. Adjusting parameters like l or b/h alters the natural frequencies of all the modes. This creates sufficient spectral separation between the bending modes and the high-frequency components of the drive signal. Consequently, the bending modes are no longer excited by the drive signal, effectively suppressing the mode coupling phenomenon at its source.

The relationships shown in Figure 7 can be utilized to select structural parameters that minimize the quadratic nonlinear coefficient k_1_. This proactive design-stage optimization eliminates the root cause of the trajectory deviations caused by mode coupling, significantly improving the accuracy of trajectory detection systems that rely on sinusoidal assumptions, without requiring complex post-fabrication compensation techniques.

## 6. Conclusions

The studied micromirror exhibited measurable deviations between the actual vibration trajectory and the ideal sinusoidal pattern. The deviations significantly degraded the trajectory prediction accuracy of the detection system. A systematic investigation was conducted to identify the origin of these deviations. Actuated by the square-wave signal, the fundamental frequency component activated the torsional-mode vibration, whereas the high-frequency component simultaneously excited the in-plane and out-of-plane bending modes, resulting in mode coupling. Considering the mode coupling effects, the coupled nonlinear term in the torsional mode vibration equation was derived through Hamilton’s principles. The vibration equation was solved numerically to predict the vibration trajectory. Both simulated and experimental analyses confirmed that the mode coupling effects generated the second harmonic component in the vibration trajectory spectrum, and the component constituted the primary source of the observed deviations. In order to improve the accuracy of the trajectory detection system, a design-stage optimization framework that tunes structural parameters to achieve spectral separation between the bending mode and the torsional mode is proposed, thereby suppressing the mode-coupling-induced nonlinearity.

## Figures and Tables

**Table 1:** The main structural parameters of the micromirror.

Parameter	Value	Unit
Mirror plate radius, R	1.5	mm
Torsion beam width, b	18	μm
Torsion beam length, l	400	μm
Movable comb beam width, B	265	μm
Movable comb beam length, L	0.9	mm
Device thickness, h	50	μm

**Table 2:** Parameters of torsional-mode vibration equation.

Parameter	Value	Unit
Inertia, J	4.64 × 10^−13^	Kg·m^2^
Torsional stiffness, k	2.35 × 10^−5^	N·m·rad^−1^
Damping coefficient, c	1.33 × 10^−11^	N·m·s·rad^−1^
Quadratic nonlinear coefficient, k_1_	5.70 × 10^−6^	N·m·rad^−2^
Coefficient parameter of drive torque, b_0_	4.07 × 10^−12^	F
Exponential parameter of drive torque, b_1_	48.81	rad^−1^
Square-wave voltage amplitude, U_m_	120	V

## Data Availability

The raw data supporting the conclusions of this article will be made available by the authors on request.
